# Bioprocess monitoring: minimizing sample matrix effects for total protein quantification with bicinchoninic acid assay

**DOI:** 10.1007/s10295-016-1796-9

**Published:** 2016-06-17

**Authors:** Wieland N. Reichelt, Daniel Waldschitz, Christoph Herwig, Lukas Neutsch

**Affiliations:** 1 Christian Doppler Laboratory for Mechanistic and Physiological Methods for Improved Bioprocesses, Institute of Chemical Engineering, Vienna University of Technology, Getreidemarkt 9/166, 1060 Vienna, Austria; 2 Research Division Biochemical Engineering, Institute of Chemical Engineering, Vienna University of Technology, Gumpendorfer Strasse 1A/166-4, 1060 Vienna, Austria

**Keywords:** Total protein measurement, Bioprocess analytics, BCA measurement in complex sample matrix, BCA assay interference, TCA protein precipitation

## Abstract

**Abstract:**

Determining total protein content is a routine operation in many laboratories. Despite substantial work on assay optimization interferences, the widely used bicinchoninic acid (BCA) assay remains widely recognized for its robustness. Especially in the field of bioprocess engineering the inaccuracy caused by interfering substances remains hardly predictable and not well understood. Since the introduction of the assay, sample pre-treatment by trichloroacetic acid (TCA) precipitation has been indicated as necessary and sufficient to minimize interferences. However, the sample matrix in cultivation media is not only highly complex but also dynamically changing over process time in terms of qualitative and quantitative composition. A significant misestimation of the total protein concentration of bioprocess samples is often observed when following standard work-up schemes such as TCA precipitation, indicating that this step alone is not an adequate means to avoid measurement bias. Here, we propose a modification of the BCA assay, which is less influenced by sample complexity. The dynamically changing sample matrix composition of bioprocessing samples impairs the conventional approach of compensating for interfering substances via a static offset. Hence, we evaluated the use of a correction factor based on an internal spike measurement for the respective samples. Using protein spikes, the accuracy of the BCA protein quantification could be improved fivefold, taking the BCA protein quantification to a level of accuracy comparable to other, more expensive methods. This will allow reducing expensive iterations in bioprocess development to due inaccurate total protein analytics.

**Graphical abstract:**

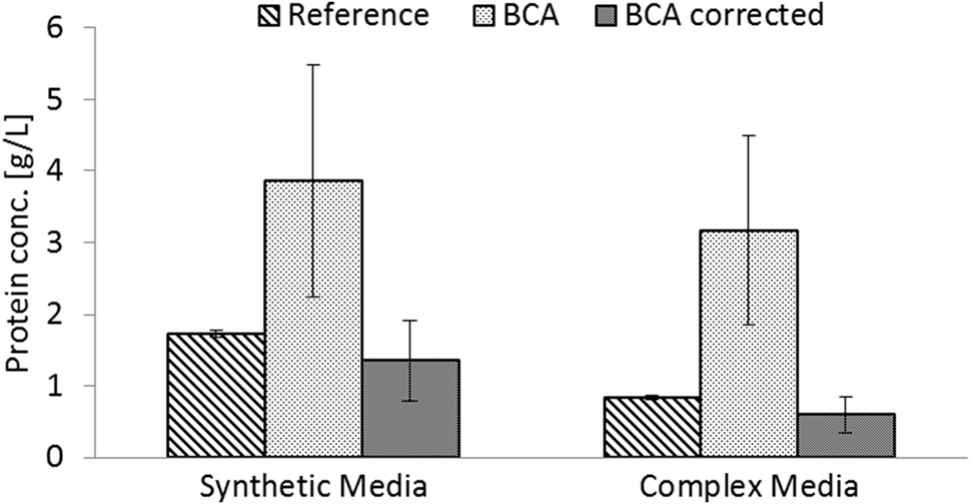

**Electronic supplementary material:**

The online version of this article (doi:10.1007/s10295-016-1796-9) contains supplementary material, which is available to authorized users.

## Introduction

In biotechnology and numerous other scientific areas, a precise measurement of the protein concentration is of great relevance [44]. Especially in the field of recombinant production of biopharmaceuticals and other high-value added compounds, the total protein concentration serves as a key variable for process development and quality control purposes [16–18]. The total protein release into the culture supernatant can not only give a direct estimate of productivity in case of secreted proteins but also provides valuable information on the overall cell physiology during a bioprocess. As is the case for other critical variables, the strive for deeper bioprocess understanding calls for analytical methods that are accurate, sensitive, robust and cost efficient. Despite substantial efforts to identify and remove interfering substances, complex sample matrices still put limitations on the commonly used assays for total protein quantification. Unfortunately, the biasing influence of medium components is often neglected in bioprocess monitoring and appropriate controls are not included.

Commonly, two approaches for total protein quantification are employed: non-colorimetric or colorimetric assays. Non-colorimetric measurements of the protein concentration, e.g. amino acid analysis [38], size exclusion chromatography or mass spectrometry are usually linked to a high instrumental expense and effort in preparatory work [14]. The high protein specificity of the latter methods is advantageous for target protein quantification. However, the same, high sensitivity towards different types of proteins and the associated need to use appropriate standards are significant drawbacks in the context of total protein quantification. In case of methods that are less specific, e.g. UV/Vis-based platform SoloVPE [30] instrumental advances have allowed for an increase in sensitivity and decrease of the sample volume. Nevertheless, protein quantification via UV/VIS absorption is often hindered by sample matrices containing unsaturated fatty acids [47]. In colorimetric assays, lab-on-a-chip systems [2, 14, 35] have led to substantial progress in terms of sensitivity and reproducibility. These techniques combine a chromatographic separation phase to the colorimetric detection step, leading to good resolution and sensitive quantification. However, the chromatographic separation step has to be specifically adapted to the sample matrix. In case of bioprocess samples this sample matrix can be subjected to dynamical changes over process time. This requires case-by-case adaptations of the chromatographic separation step and makes total protein quantification via such systems tedious. Additionally, owing to the need for advanced microfluidics in the chip technology, these assays are linked to substantial investments and higher consumable expenses as compared to conventional colorimetric assays.

Wet-chemical assays are more cost efficient and, although involving several handling and preparation steps, usually allow for high-throughput analysis. The underlying principle of a more or less uniform protein staining, based merely on amino acid residues, is an advantage in the context of total protein quantification. In combination with their simplicity the latter characteristics are the reason for the wide usage of these wet-chemical assays for total protein quantification [6, 13, 29, 32, 47]. Bradford, Lowry and the Bicinchoninic acid (BCA) assay are the most commonly used colorimetric assays. Especially in microbial bioprocesses the composition of the supernatant sample usually becomes increasingly complex throughout the fermentation time course, mainly due to a gradual accumulation of sugars, phospholipids, DNA and salts. Considerable research effort has been devoted to the direct comparison of the available colorimetric assays, leading to some general recommendations regarding assay usage [11, 22, 29, 37].

The Bradford or Coomassie Blue assay is based on a residue-specific stain, first described by Bradford [4]. Via hydrophobic interactions, Coomassie Brilliant Blue G-250 [11] binds to arginine, histidine, phenylalanine, tryptophan and tyrosine residues [8] at acidic pH. Disadvantages of this assay include sensitivity to different reagent formulations [33] as well as the high sensitivity to varying amino acid composition [8]. This sensitivity to the amino acid distribution renders the method less applicable for the generic quantification of the total protein content in biotechnology.

The Lowry assay is based on a two-step chemical reaction: first, a reduction of cupric ions to cuprous ions under alkaline conditions, and second, a reduction of protein residues [24]. This reduction is followed by a reaction with the Folin–Ciocalteu reagent, resulting in a blue complex absorbing at 750 nm [31]. Since the color formation is not only induced by cuprous ions, but also by chromophoric amino acids such as tyrosine, tryptophan, phenylalanine [48] as well as cysteine residues [10], differences in the content of the various amino acids can cause high protein-to-protein variation. Nonionic detergents have been reported to form a precipitate with the Folin–Ciocalteu reagent and the use of anionic detergents such as sodium dodecylsulphate (SDS) or sodium deoxycholate (DOC) has been proposed to counteract this problem [9]. Adopting the use of DOC without further investigation, a precipitation step has been found beneficial for the removal of interfering substances from artificial samples [3]. More advanced modifications of the Lowry assay have been developed to improve robustness against interfering substances, as well as linear range. Nevertheless, the assay is still being outmatched by the BCA assay in terms of linear range and sensitivity [6].

In the BCA assay, the Folin–Ciocalteu reagent is replaced with bicinchoninic acid as described by Smith [40]. Unlike the Bradford and the Lowry, the BCA assay features a relatively small protein-to-protein variation [11, 28], making it the most suitable assay for total protein quantification. As described in literature [12, 22, 44], the BCA assay is the best choice for samples with undefined protein content in the presence of detergents. Even in combination with a DOC-TCA precipitation step, the Lowry assay is outmatched by the standard BCA assay in terms of robustness towards interfering detergents [34]. This is of particular relevance when analyzing the supernatant of culture medium, which often contains significant amounts of biological (e.g. DNA and phospholipids of cellular origin) and synthetic surface-active compounds, e.g. nonionic detergents such as antifoam additives.

Several other substances are known to cause interference with the BCA measurement in bioprocess samples, including medium components like ethylenediaminetetraacetic acid (EDTA) [45], reducing sugars [5, 43] like fructose or lactose [34] and metabolites as phospholipids [19] or biogenic amines. Already the work of Smith [40] highlighted the need to implement proper controls and, if necessary, pre-treatment steps to avoid interference. Efforts have been undertaken to remove interfering substances, e.g. by precipitation with TCA [5, 26, 39]. Hereby, DOC has occasionally been used in combination with TCA [5, 39] referring to work based on the Folin–Ciocalteu reagent [3]. However, to our knowledge, up to now no statistical significant data has been provided in literature indicating the benefit of the additional use of DOC compared to the mere TCA precipitation in the context of the BCA assay. To account for interference of the sample matrix, countermeasures have been described which aim at the identification of the interfering substances [27]. Once identified, the components can be accounted for during calibration. Unfortunately, bioprocess samples often are subjected to unpredictable changes in the amount and nature of the interfering components, leading to substantial bias. Despite multiple accounts in literature pointing out the risk and impact of sample matrix interference for the BCA assay, many researchers are too confident regarding the universal applicability of this long-established standard procedure [18, 20, 25, 36].

This work presents an application-oriented re-assessment of the BCA assay as the current state-of-the-art method in bioprocess protein quantification. We demonstrate the substantial bias caused in total protein quantification when following standard protocols over the course of typical fed batch cultivations and demonstrate how simple adjustments to the method can lead to remarkable improvements in measurement accuracy,

## Materials and methods

### Media

One industrially relevant complex and one synthetic culture medium were tested in a typical fed batch bioprocess [23]. *Escherichia coli* was cultivated at controlled pH (7), DO_2_ (>30 %) and temperature (30 °C) to high cell density (biomass concentration >40 g/L). In the complex medium the *E. coli* strain K12 was grown with glycerol as C-source, producing a Fab antibody as soluble intracellular protein (~24 kDa) throughout an induction phase of 48 h. The complex medium was based on the formulation given in Wilms et al. [46], supplemented with complex medium components. In the synthetic medium, based on the formulation of Korz et al. [21] the *E. coli* strain BL21 DE3 was grown on glucose as a C-source. During induction phase an intracellular protein (~30 kDa) was expressed which led to the formation of inclusion bodies.

### Samples

Time-resolved fermentation samples were taken throughout induction phase of the experiments and labeled from A-I. The samples were cleared from cells and other debris (10,000 rpm; 10 min, 4 °C). The clear supernatant served as sample for further investigation and was stored at −20 °C.

### Trichloroacetic acid (TCA) precipitation

Prior to protein quantification by BCA assay, the protein was isolated via TCA precipitation [42]. 500 µL of 10 % TCA solution (Carl Roth, Austria, 8789) in MilliQ was added to 500 µl of sample. After 10 min incubation on ice the samples were centrifuged (10,000 rpm; 10 min, 4 °C). Subsequently, the supernatant was discarded and the pellet re-dissolved in 1 mL of the reference sample buffer 0.1 M NaOH/1 % SDS (NaOH/SDS).

### BCA assay

Using a commercial BCA assay kit (Sigma, Austria, B9643) assay according to [40] the samples were incubated at 60 °C for 15 min to ensure the lowest protein-to-protein variations. After incubation, the samples were equilibrated for 10 min at room temperature prior to absorbance measurement within the linear range from 0.1 to 0.7 relative absorption units (rAU). The correlation between signal and protein concentration was established based on a separate calibration from 0.05 to 1 g/L BSA in NaOH/SDS. The limit of detection (LOD) was determined at 0.2 g/L.

### Protein spiking

In contrast to “uncorrected” native samples the “spiked” samples were spiked with bovine serum albumin (BSA) (Carl Roth, Austria, 3737, >98 % purity, IgG and protease free) in a concentration range of 0–10 g/L. Two different spike levels were used to correct for matrix effects in the BCA assay (detailed below). In brief, each sample was diluted 1:1 with a BSA stock solution (1000 or 500 µg/mL) after the TCA precipitation step. Four different sample dilutions (in NaOH/SDS 1:4, 1:8, 1:16 and 1:32) were analyzed in each run. The protein concentrations were calculated from the mean values of the repetitive measurements. The number of replicates is indicated in each section. The quotient of measured and theoretic spike concentration was calculated to serve as a correction factor. For quantitative evaluation of the method accuracy, the relative deviation of corrected/uncorrected protein concentrations from the reference protein concentration was determined. Reference protein quantification is described below (“Quantification of total nitrogen”).

### Efficiency assessment of the TCA precipitation step

The BSA standard used for the spikes was supplemented (1:100) with FITC-labeled fBSA (Sigma, Austria, A9771). Fluorescence was measured with an Infinite M200 plate reader (Tecan Group Ltd) in a dilution of the sample 1:10 with NaOH/SDS in 96 multiwell plates (M&B Stricker, Germany, GRE-655101) with an excitation wavelength of 485 nm and an emission wavelength of 525 nm. The fluorescence signal of the samples before precipitation was compared to the fluorescence signal of the precipitated and re-suspended sample.

### BCA assay corrected with one spike level

All samples were spiked with 500 µg/mL BSA. The average was calculated from triplicate measurements within the linear range. To account for the effect of matrix components, the measured protein concentration of the unspiked samples was subtracted from the measured protein concentration of the spiked samples to determine the contribution of the added spike (Eq. 1). The quotient of theoretic and measured spike concentration served as correction factor (Eq. 2) of the measured protein concentration of each sample (Eq. 3).

### BCA assay corrected with two spike levels

All samples were spiked (see “Protein spiking”) separately with 250 and 500 µg/mL BSA. All measurements (incl. dilutions) were measured in triplicates. The correction factor *k* corresponds to the slope of the correlation of measured and theoretic concentrations of 0/250/500 µg/mL spikes. *k* was calculated separately for each sample and for each dilution (Eq. 2). Finally, the mean of the corrected protein concentration calculated over all dilutions within the linear range, yielded the final protein concentration.

1 $$C_{\text{sm}} = C_{\text{ps}} - C_{\text{p}}$$

The measured spike concentration (*c*_sm_) is derived from the measured protein concentration of the spiked sample (*c*_ps_) and the measured protein concentration of the unspiked sample (*c*_p_).

2 $$\frac{{C_{\text{st}} }}{{C_{\text{sm}} }} = k$$

Accounting for matrix effects with two spike levels. The theoretic spike concentration (*c*_st_) correlates to the measured spike concentration (*c*_sm_) by the factor (*k*). In case of one spike (*k*) is simple a proportionality factor. In case of two spikes (*k*) corresponds to the slope of the correlation (0/250/500 µg/mL BSA) of *c*_st_ and *c*_sm_ for the utilized spike concentrations.

3 $$C_{\text{pc}} = C_{\text{p}} \times k$$

The corrected protein concentration (*c*_pc_) is calculated from the measured protein concentration of the unspiked sample (*c*_p_) and the correction factor (*k*).

### Quantification of total nitrogen (TN)

For verification purposes, measurements of the total nitrogen bound (TN) were conducted. The total nitrogen content was quantified by an adapted method based on peroxodisulfate oxidation of nitrogen compounds in water to nitrate, with consequent detection with copperized cadmium according to DIN EN ISO 11905-1 (Technical Committee ISO/TC 147 [41]. Samples were pre-diluted to an approximate concentration of 5–50.00 mg/mL total nitrogen. The LOD of the method was determined at 5.27 mg/L total nitrogen. Data below the LOD were set to 0 mg/L. According to a calibration (Supplemental 1) with BSA as standard protein the total protein content of the sample was calculated based on the TN content of each sample.

### Statistical data analysis

Data were subjected to statistical analysis (2 sample *F* test, 2 sample *t* test, Welch test) Datalab Version 3.5 (distributed by Epina http://datalab.epina.at/). Based on an *α* = 0.05 the significance of the correlation was evaluated at hand of the *p* value.

## Results

### BCA-based protein quantification is significantly impacted by sample matrix composition

**Fig. 1:**
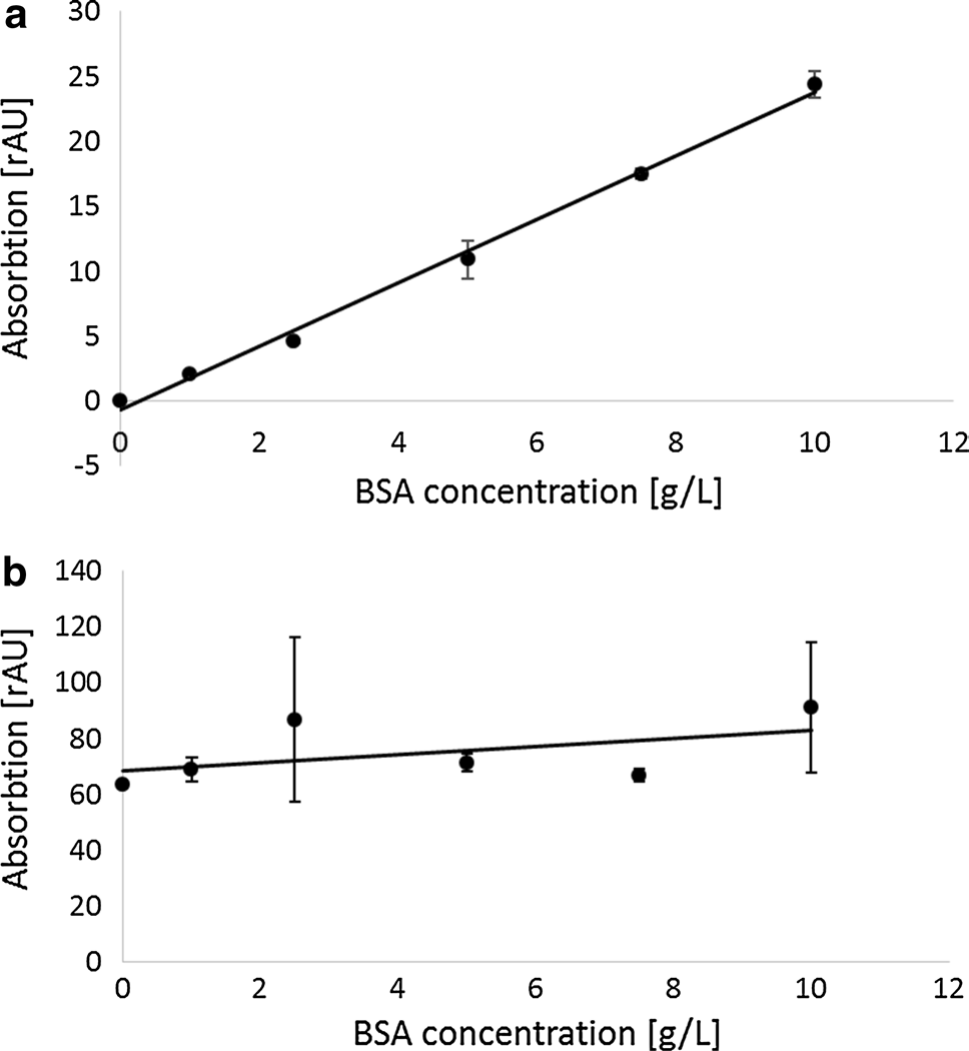
Protein quantification by BCA assay is highly sensitive to sample matrix composition; protein quantification of BSA dilution rows in reference buffer versus spent synthetic culture medium as sample matrix. The standard deviations for each sample (*n* = 5) are indicated as *whiskers*; **a** dilution row of BSA measured in the background NaOH/SDS yielding a *R* ^2^ of 0.995 and a mean relative standard deviation of 5.43 %. **b** Dilution row of BSA standards measured in synthetic culture medium yielding a *R* ^2^ of 0.255 and a mean relative standard deviation of 12.6 %. In fermentation medium the 10 g/L spike signal is not significantly larger than the 1 g/L spike level (Welch test: *p*(*t*) = 0.0796)

To demonstrate the impact of sample matrix interference, a dilution row of bovine serum albumin (BSA) was prepared in reference buffer NaOH/SDS (Fig. 1a) and in fermentation supernatant (Fig. 1b). Measuring the concentrations of BSA in the background of NaOH/SDS via the BCA assay yielded highly accurate results. This confirms the general capability of the BCA method to quantify total protein with high reproducibility under ideal conditions. However, if synthetic *E*. *coli* culture medium from actual process samples was used as matrix, it was not possible to resolve differences in protein concentration up to 10 g/L.

### Quantitative protein precipitation by TCA

**Fig. 2:**
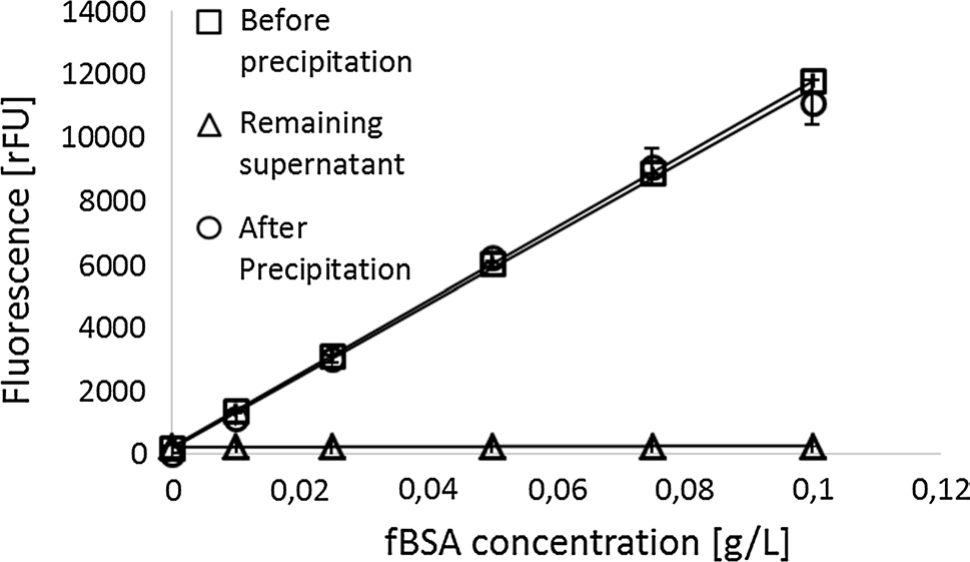
Protein precipitation by TCA is quantitative; fluorescence measurements of BSA dilution rows of TCA-precipitated, synthetic process media supplemented with fBSA are displayed. All samples were measured in quadruplicates (*n* = 4); the standard deviations are indicated as *whiskers*. 99.7–97.5 % of the added fBSA was recovered in the reference buffer (after precipitation), and only 0.3–2.5 % of the initial fluorescence was found in the supernatant (remaining supernatant). The fluorescence intensity before precipitation (before precipitation) and after precipitation (after precipitation) correlated with the nominal concentration of the stock solution (*R* ^2^ > 0.99). The fluorescence intensity found in the supernatant is almost negligible and not correlated with the spike concentration (*R* ^2^ = 0.24)

The lack in sensitivity of the BCA protein quantification method in complex sample matrixes (Fig. 1) may be improved by removal of the interfering substances and error compensation. The basic aim of introducing a precipitation step is to remove interfering substances from the sample matrix. While interfering substances should be retained in the supernatant, protein shall be quantitatively precipitated in the pellet. Commonly, such matrix replacement is performed by TCA precipitation, followed by re-suspension in a defined reference buffer such as NaOH/SDS. However, to reliably exclude measurement bias caused by the precipitation step itself, the efficiency of the TCA precipitation procedure first has to be evaluated. To this end, a dilution row of BSA in fermentation supernatant was additionally supplemented with a defined amount of fBSA, which allows for identifying potential protein loss during the workup procedure via direct fluorescence readout. Samples were precipitated, the pellets dissolved in fresh buffer and all resulting fractions were analyzed for fluorescence intensity (Supplemental 2). 99.7–97.5 % of the added fBSA was recovered after precipitation in the reference buffer (Fig. 2) from culture medium supernatant 48 h post-induction. Native medium and medium 24 h post-induction gave the same results (data not shown). Based on the results it can be concluded that the precipitation of protein by TCA is highly efficient and unlikely to cause significant measurement bias due to uncontrolled loss of protein.

### Interfering substances accumulate in the culture medium

**Fig. 3:**
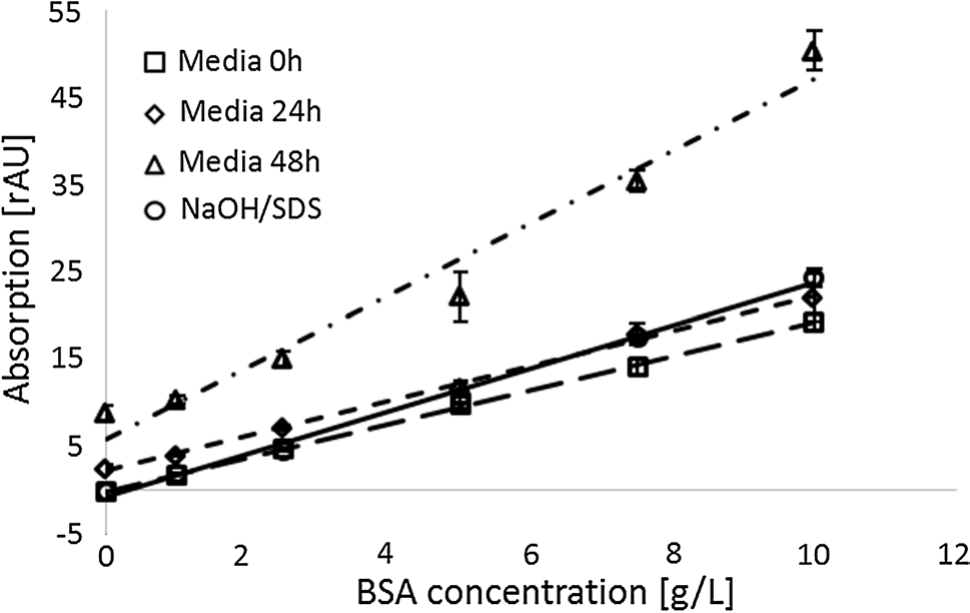
The impact of interferences increases over process time and traces back to cell-related processes: BSA spikes (concentrations 0–10 g/L) were added to precipitated fermentation samples obtained from different time points of a cultivation performed in synthetic medium. Comparison to BSA concentrations measured in reference buffer NaOH/SDS. For late time points (48 h) of the fermentation, the correlation of signal-to-protein is altered substantially. All samples were measured in quadruplicates (*n* = 4); the standard deviations are indicated as *whiskers*

After substantiating the quantitative precipitation of protein by TCA (Fig. 2) the potential origin of the observed interference on BCA assay readout was investigated in further detail. In principle interfering substances may originate from the biomass or be contained in the medium formulation. To elucidate the basic root cause of the interference, the supernatant of precipitated culture medium from 0/24/48 h after induction was subjected to total protein determination. Equal dilution rows of BSA were prepared in the supernatant of TCA-precipitated culture medium. As shown in Fig. 3, no interfering substances seemed to accumulate in the supernatant over the first 24 h of the process, since the signal-to-protein correlation was not significantly altered in comparison to the reference buffer. However, a clear change in signal correlation became visible after a process time of 48 h. Regardless of the identity of the interfering substances present in spent culture medium, their persistent biasing effect has to be accounted for.

### TCA precipitation alone is not sufficient to avoid interference

**Fig. 4:**
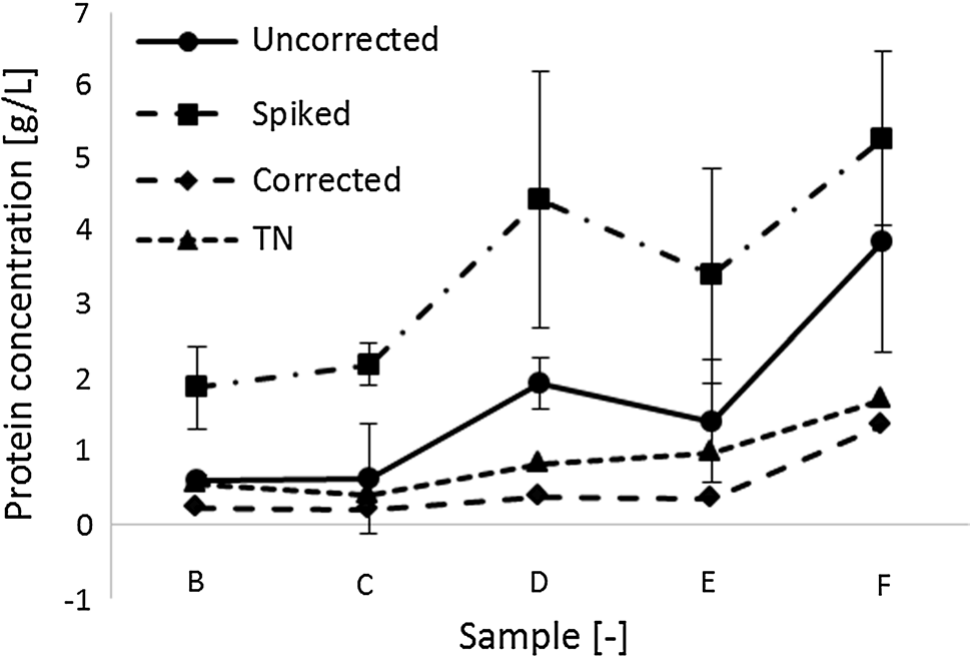
Correction of protein determination based on spike addition leads to an increase in accuracy: samples from consecutive time points during the fermentation in synthetic medium between 0 and 24 h after induction (*B*–*F*). All measurements were performed after TCA precipitation. *uncorrected* measured protein concentration of native samples; *spiked* measured protein concentration of samples with spike (500 µg/mL); *TN* measured reference protein concentration derived from TN based protein quantification; *corrected* calculated protein concentrations calculated according to Eq. 3. *Lines* between measurement points have been included to ease orientation. The relative differences of the corrected protein concentration from the TN derived protein concentrations are significantly smaller than the respective relative differences of the uncorrected concentrations [*p*(*t*) = 0.008]. The relative standard deviation of the respective differences is for the corrected values (16 %) significantly [*p*(*F*) = 0.004] smaller than of the relative uncorrected protein concentration (85 %). BCA protein quantification was performed in triplicates (*n* = 3); the mean values were used for calculation. The standard deviation is indicated as *whiskers*

**Fig. 5:**
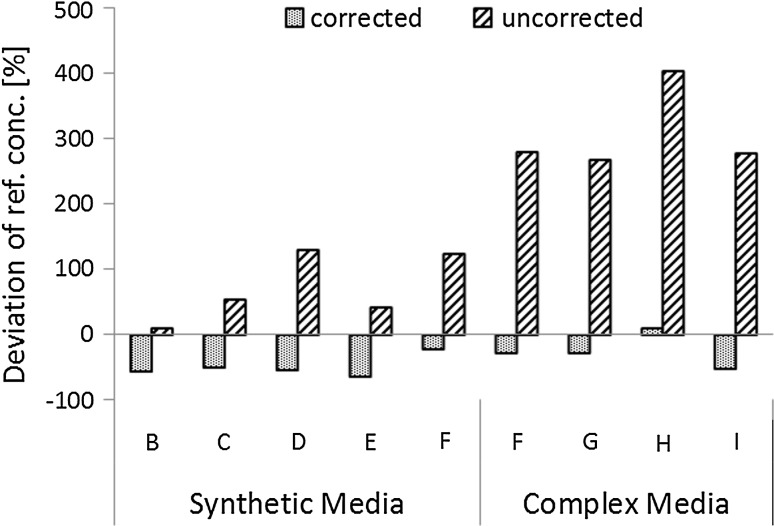
Relative error of measurement is reduced from 212 to 41 % in average by the use of one spike: samples from consecutive time points during the fermentation in a complex and a synthetic culture medium. The *letters B*–*I* refer to different time points during the fermentation. Differences of protein concentrations derived from BCA measurements (*corrected*/*uncorrected*) compared to protein concentrations according to TN method are plotted on the *y* axis [deviation from ref. conc. (%)]. The relative differences of the corrected protein concentration (41 %) from the TN derived protein concentrations are significantly smaller [*p*(*t*) = 0.0001] than the respective relative differences of the uncorrected concentrations. The standard deviation of these respective differences is for the corrected values (14 %) significantly smaller [*p*(*F*) = 0.0000] than the standard deviation for the uncorrected protein concentration (127 %). All values under the LOD of the TN measurement of 5.27 mg/L were set to zero and are not displayed. All samples were measured in triplicates (*n* = 3), the mean values were used for calculation

**Fig. 6:**
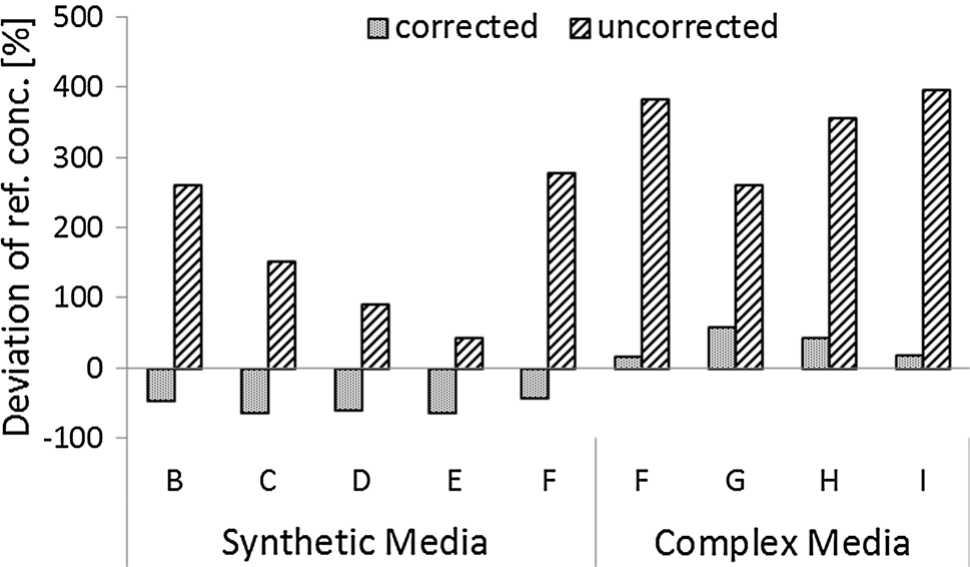
Relative error of measurement is reduced from 212 to 46 % in average by the use of two spikes: Samples from consecutive time points during the fermentation in a complex and a synthetic culture medium. The *letters B*–*I* refer to different time points during the fermentation. Differences of protein concentrations derived from BCA measurements (*corrected*/*uncorrected*) compared to protein concentrations according to TN method are plotted on the *y* axis [deviation from ref. conc. (%)]. The relative differences of the corrected protein concentration (46 %) from the TN derived protein concentrations are significantly smaller than the respective relative differences of the uncorrected concentrations [*p*(*t*) = 0.0001]. The standard deviation of these respective differences is for the corrected values (17 %) significantly smaller [*p*(*F*) = 0.0001] than the standard deviation for the uncorrected protein concentration (112 %). All values under the limit of detection (LOD) of the TN measurement of 5.27 mg/L were set to zero and are not displayed. All samples were measured in triplicates (*n* = 3) and the mean values were used for calculation

A constant impact of the interfering substances, without changes over process time, would permit straightforward correction of the BCA measurements via a given, pre-defined factor. It was thus important to check in how far the distortion of the signal-to-protein ratio changes over process time (*B*–*F*). The BCA analysis was compared to total nitrogen measurements (TN) as an orthogonal method for protein quantification (Fig. 4). However, the correlation between the uncorrected protein concentration obtained via the BCA measurement and the protein concentration determined via TN changed over process time, substantiating the need for a sample-specific compensation strategy. The direct comparison of the uncorrected protein concentrations derived from TCA-precipitated samples to the reference protein concentration (TN) yielded an enormous average relative deviation of 212 % (Figs. 4, 5, 6).

It can thus be concluded that TCA precipitation only is an insufficient strategy to avoid sample matrix interferences in bioprocess analysis and additional corrective actions are required to avoid misestimation of total protein content. By individual spiking of each sample the process time-dependent impact of matrix components on TCA-precipitated samples can be corrected (Fig. 4). Despite overcompensation, the correction led to a substantial increase in convergence of the BCA assay derived protein concentrations and the actual protein concentration (TN).

Having established the qualitative benefit of corrections via spike addition (Fig. 4), a quantitative evaluation was the next step to conclude on the practical usability of the modified protocol. In order to prove the generic applicability, we tested the approach for two different medium formulations. Interestingly, in complex medium the apparent total protein concentration in [g/L] was found to be in average two- to threefolds higher as compared to synthetic medium (data not shown). Figure 5 displays the deviation of the uncorrected and corrected protein concentrations from the protein concentrations derived from TN measurement. By correcting the values of the unknown samples according to Eq. 3, the deviance was substantially reduced from 212 to 41 % for synthetic medium as well as for complex medium. Moreover, the method error became significantly more systematic, with the variance in deviation decreasing from 127 % to 14 % for both options.

The results shown in Fig. 5 led to the question whether assay accuracy could be further improved by the use of an additional spike level. The benefit of measuring two internal spike levels per sample is exemplified in Fig. 6. Using two spikes, the deviation was reduced to 45 % in respect to the uncorrected values. However, in case of the synthetic medium the relative deviations of the uncorrected protein quantification declined over time in contrast to the trajectory of the deviations for one spike level. This may be attributed to the generally low protein concentrations for the strain grown in synthetic medium. Especially samples B and C displayed protein concentrations close to the limit of detection of the BCA assay.

The overestimation of protein content for the corrected values can presumably be attributed to dilution effects, which may in this case be more severe owing to the genuinely higher protein concentrations in complex medium. In comparison to one spike level, the dynamic range of the assay did not allow the measurement of the native sample and the two different spike levels within one dilution. Two spikes yielded a variance of deviation not significantly smaller [*p*(*F*) = 0.207] than for one spike. Concluding, the use of two spike levels does not lead to any significant improvements in terms of measurement accuracy.

## Discussion

Far too often, a widely used standard procedure like the BCA or Bradford assay is adopted in the erroneous assumption that straightforward method transfer between different applications is possible. Bioprocess samples are especially challenging in this regard. Routine biotechnological monitoring strategies typically cover a time series analysis of multiple consecutive samples over the duration of the process. A plethora of uptake and secretion and release processes related to metabolic turnover, as well as time-dependent cellular lysis can lead to substantial, yet gradually evolving changes in the chemical composition of the culture supernatant. If one or several of the changing factors happen(s) to have an impact on measurement accuracy, this biasing effect(s) too will evolve gradually without brisk steps being visible in the signal-over-time-curve. Many researchers tend to focus on the smoothness of measurement values over time as a primary indicator for data quality and hence will fail to detect such errors. The required, thorough method qualification is frequently omitted for sake of time, regardless of the important role of total protein determination in bioprocess engineering. Total protein often serves as the key variable to conclude on culture physiology, and critical decisions in process development as well as strain screening are based on the protein data. In at-line process monitoring, the determination of the ideal point of harvest or detection of unintended cell lysis events rely on a robust method for protein quantification.

Unfortunately, even if respective controls at early and late process times are being included, a negative check for interfering substances has to be repeated as soon as any major change is brought to the process setup that may lead to a change in matrix composition. This would lead to significant complications in the usual, iterative workflow of process optimization. There is thus a substantial need for refined analytical protocols that allow for taking such factors into account, yet without increasing operator workload beyond a reasonable extent. Against this backdrop, the aim of this study was to evaluate and to illustrate the impact of matrix components on protein quantification by the means of the BCA assay, as well as the elaboration of a rapid and generally applicable method to compensate for the biasing effects.

In our studies, the original BCA assay was found incapable of resolving an addition of up to 10 g/L BSA in complex sample matrices. This substantial loss in sensitivity underlines the necessity to remove interfering substances, as it has been advised in the past [5, 8]. This shortcoming of the standard BCA assay has also been reported recently in other context [47], albeit in this case assay performance could be remarkably improved via TCA precipitation. This was not the case for the systems investigated here, as well as for several other bioprocess setups that were evaluated in our and other laboratories (personal communication to the authors). We hence speculate that a considerable number of biotechnology R&D projects will experience similar problems, often without being aware of it.

One potential cause for the failure of the TCA protocol to improve measurement consistency in case of the investigated bioprocess media could lie in a changing efficiency of the protein denaturation, precipitation or re-solubilisation step. We were, however, able to show that the loss of protein is far too low to account for the observed bias. Also a standardization of the pH value after TCA precipitation, which is a known cause for variations in the dye-protein reaction [40], was found to remain without consequences for signal quality in the present case. Several modifications and fine-adjustments of the TCA protocol, including wash or solubilization steps with pH-stabilizing reagents such as NaOH or HCl, that were successfully employed in other settings [5], evidently could not remove the source of bias in complex culture medium. It should be noted in this regard that an interplay of multiple biasing substances, may account for the observed interference, which is why wash protocols from more defined applications may fail. Also others have reported such continuing interference after TCA precipitation, but in this case acid wash let to a substantial reduction of interferences [39].

The dynamically changing impact of sample matrix components, illustrated in this work, is indicative of a highly complex matrix composition, presumably not only in terms of concentration but also regarding the chemical nature of the individual agents. Given the complete lack of knowledge on type and amount of the interfering agents, it would be risky and probably counterproductive to include time-intensive purification protocols (e.g. by dialysis) in the workup chain, as was proposed for BCA assays when applied to bioprocess monitoring [27]. In direct comparison to TN quantification as a reference method, the spike-corrected BCA measurement protocol led to a systematic underestimation of the protein concentration. Although the correction leads to an underestimation of the protein concentration, it yields more accurate and reproducible values for all tested strains in all tested media. The systematic underestimation may partially trace back to differences in both methods, regarding the sensitivity for the BSA standard, since, depending on the molecular weight, the average nitrogen content in proteins found in the culture supernatant may differ from the nitrogen content of BSA [7]. Principally, such deviations could be corrected for via an error offset that could be determined for each fermentation run by parallel analysis of chosen samples via the TN method. However, a prerequisite for this correction approach would be a constant nitrogen level in the supernatant, which may be critical especially in processes where NH4 is used for pH correction after acetate production.

Other methodological alternatives proposed for total protein quantification in fresh and complex cell culture media include fluorescence anisotropy as proposed by Groza et al. [15]. However, sensitivity of the method in the dynamic environment of microbial bioprocesses was not investigated up to now and remains to be demonstrated. In comparison to the relative standard deviation of 15–46 % in BSA protein quantification achieved with a bioanalyzer© system [1], the relative standard deviation of 41 % for process samples obtained via the herein proposed improved measurement protocol is within an attractive range. Especially with regard to the limited effort for data processing and instrumental costs, the BCA assay appears highly suited for a broad range of applications. In conclusion, the proposed method of compensation renders the BCA assay a highly cost- and time-efficient method for total protein quantification in complex sample matrices. In the context of bioprocess monitoring and development, the refined approach can be expected to help to improve existing control strategies and reduce the effort in development iterations.

## Electronic supplementary material

Below is the link to the electronic supplementary material.

**Supplemental 1** Correlation of total nitrogen (TNb) and BSA in the Range of 0–1000 mg/L (TIFF 56 kb)

**Supplemental 2** Experimental procedure of the fluorescence measurements of BSA dilution rows of TCA-precipitated samples supplemented with fBSA. (TIFF 60 kb)
