# Supplementary figures and images for: Bioprocess monitoring: minimizing sample matrix effects for total protein quantification with bicinchoninic acid assay
Source: J Ind Microbiol Biotechnol. 2016 Jun 17;43:1271–80. doi: 10.1007/s10295-016-1796-9 (PMC4983285; doi:10.1007/s10295-016-1796-9)

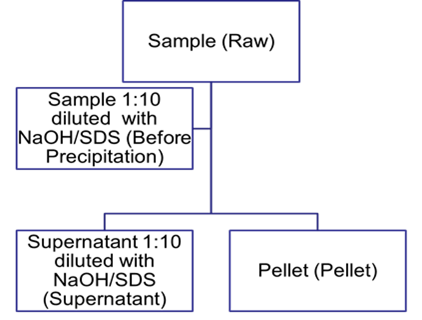

Supplement: Supplementary file 1 — Supplemental 1 Correlation of total nitrogen (TNb) and BSA in the Range of 0–1000 mg/L (TIFF 56 kb) [file 10295_2016_1796_MOESM1_ESM.tif]

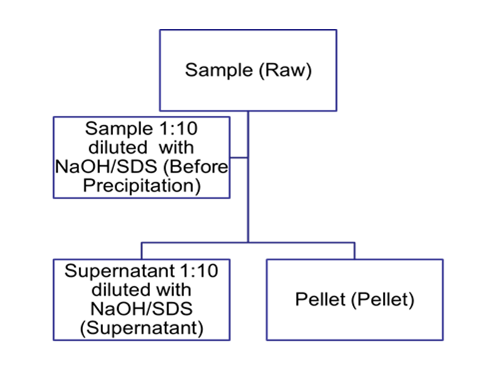

Supplement: Supplementary file 2 — Supplemental 2 Experimental procedure of the fluorescence measurements of BSA dilution rows of TCA-precipitated samples supplemented with fBSA. (TIFF 60 kb) [file 10295_2016_1796_MOESM2_ESM.tif]
